# Your Robot Therapist Will See You Now: Ethical Implications of Embodied Artificial Intelligence in Psychiatry, Psychology, and Psychotherapy

**DOI:** 10.2196/13216

**Published:** 2019-05-09

**Authors:** Amelia Fiske, Peter Henningsen, Alena Buyx

**Affiliations:** 1 Institute for History and Ethics of Medicine Technical University of Munich School of Medicine Technical University of Munich Munich Germany; 2 Department of Psychosomatic Medicine and Psychotherapy Klinikum rechts der Isar at Technical University of Munich Munich Germany

**Keywords:** artificial intelligence, robotics, ethics, psychiatry, psychology, psychotherapy, medicine

## Abstract

**Background:**

Research in embodied artificial intelligence (AI) has increasing clinical relevance for therapeutic applications in mental health services. With innovations ranging from ‘virtual psychotherapists’ to social robots in dementia care and autism disorder, to robots for sexual disorders, artificially intelligent virtual and robotic agents are increasingly taking on high-level therapeutic interventions that used to be offered exclusively by highly trained, skilled health professionals. In order to enable responsible clinical implementation, ethical and social implications of the increasing use of embodied AI in mental health need to be identified and addressed.

**Objective:**

This paper assesses the ethical and social implications of translating embodied AI applications into mental health care across the fields of Psychiatry, Psychology and Psychotherapy. Building on this analysis, it develops a set of preliminary recommendations on how to address ethical and social challenges in current and future applications of embodied AI.

**Methods:**

Based on a thematic literature search and established principles of medical ethics, an analysis of the ethical and social aspects of currently embodied AI applications was conducted across the fields of Psychiatry, Psychology, and Psychotherapy. To enable a comprehensive evaluation, the analysis was structured around the following three steps: assessment of potential benefits; analysis of overarching ethical issues and concerns; discussion of specific ethical and social issues of the interventions.

**Results:**

From an ethical perspective, important benefits of embodied AI applications in mental health include new modes of treatment, opportunities to engage hard-to-reach populations, better patient response, and freeing up time for physicians. Overarching ethical issues and concerns include: harm prevention and various questions of data ethics; a lack of guidance on development of AI applications, their clinical integration and training of health professionals; ‘gaps’ in ethical and regulatory frameworks; the potential for misuse including using the technologies to replace established services, thereby potentially exacerbating existing health inequalities. Specific challenges identified and discussed in the application of embodied AI include: matters of risk-assessment, referrals, and supervision; the need to respect and protect patient autonomy; the role of non-human therapy; transparency in the use of algorithms; and specific concerns regarding long-term effects of these applications on understandings of illness and the human condition.

**Conclusions:**

We argue that embodied AI is a promising approach across the field of mental health; however, further research is needed to address the broader ethical and societal concerns of these technologies to negotiate best research and medical practices in innovative mental health care. We conclude by indicating areas of future research and developing recommendations for high-priority areas in need of concrete ethical guidance.

## Introduction

Research in embodied artificial intelligence (AI) has increasing clinical relevance for therapeutic applications in mental health services, that is, in psychiatry, psychology, and psychotherapy. Innovations range from ‘virtual psychotherapists’ [1] to social robots in dementia care and autism disorder [2] and robots for sexual disorders [3]. Increasingly, artificially intelligent virtual and robotic agents are not only available for relatively low-level elements of mental health support, such as comfort or social interaction, but also perform high-level therapeutic interventions that used to be offered exclusively by highly trained, skilled health professionals such as psychotherapists [4]. Importantly, such ‘virtual’ or ‘robotic therapists’ include an artificially intelligent algorithm that responds independently of any expert human guidance to the client or patient through a virtually embodied presence, such as a face icon, or a physically embodied presence, such as a robotic interface. As such, these emerging applications are distinct from the many varieties of Web-based therapy, which usually involve either a human therapist, albeit remotely (telemedicine), or the patient herself, working independently with manuals, questionnaires, or other self-help materials [5].

Embodied AI applications in mental health care carry hopes of improving quality of care and controlling expenditure [6]. In addition, they also hold the promise of reaching underserved populations in need of mental health services and improving life opportunities for vulnerable groups. However, there is a persistent gap between current, rapid developments in AI mental health and the successful adoption of these tools into clinical environments by health professionals and patients. In addition, it has been demonstrated that the interventions are often designed without any explicit ethical considerations [7]. Furthermore, although studies often examine the effectiveness or ethical use of a single application, rarely do they consider the implications for the integration of AI across the field of mental health more broadly. In this paper, we argue that virtually and physically embodied artificially intelligent agents and applications have great potential in mental health care. However, their societal and ethical implications require further probing to identify pertinent concerns surrounding trust, privacy, and autonomy, as well as to anticipate concerns that may arise in the future. Identifying the broader ethical and societal implications of embodied AI is crucial for negotiating best research and medical practices in innovative mental health care. We conclude by indicating areas of future research and identifying points in need of ethical caution.

### Overview: Existing Embodied Intelligent Applications

Although AI-enabled virtual and robot therapy has long been used across a number of medical fields [8-10], the integration of AI through the use of embodied agents is still at an early stage in mental health care; it is arguably the most recent addition to psychotherapeutic practice, supporting a host of emotional, cognitive, and social processes [11]. In what follows, we have sketched a range of applications with the aim of characterizing some of the embodied artificially intelligent innovations across the field of mental health. To maintain focus amid a broad and growing field, we have chosen to exclude from our analysis applications that are not intended to interact with patients, or that have no virtual presence or robotic interface; this includes AI-supported scanning and diagnostic tools. We have also excluded applications that may have a virtual or robotic interface but do not employ AI, such as telemedicine therapy (for further scholarship on this topic, please see [12-19]).

#### Virtually Embodied Artificially Intelligent Agents

AI-supported virtually embodied psychotherapeutic devices are currently developing at a rapid speed. For example, therapeutic apps such as Tess and other “chatbots” such as Sara, Wysa, and Woebot, which work over short message service text messaging, WhatsApp, or internet platforms, are being explored for addressing depression and anxiety. These applications come with interactive screen presences. Woebot and other programs engage with the patient like a virtual psychotherapist, with the aim of helping patients to recognize their emotions and thought patterns and to develop skills such as resilience or techniques for reducing anxiety. For example, using natural language processing, Tess is programed to flag expressions that indicate emotional distress. Often cited as a digital tool to reach underserved populations across the world that lack mental health services, the bots can explain to users the clinical terms for what they are experiencing—such as cognitive distortions—or provide concrete advice for recognizing and dealing with difficult situations [20]. Initial studies found that depression symptoms decreased with the use of Woebot more than groups who relied on electronic book resources [21], and another study found that Tess helped to reduce depression and anxiety among users [20].

A similar approach involves the use of avatars, such as the Avatar Project, for addressing persistent auditory hallucinations for patients with psychosis [22]. These usually involve computer-generated images of faces on computer screens or tablets that interact with a patient via intelligent algorithms. Avatars are also being explored in treatment of schizophrenia, for example, to improve medication adherence [23]. Similar to the Avatar Project, virtual reality–assisted therapy for schizophrenia often encourages patients to engage with the voices they hear through the use of an AI avatar. Initial studies found that the therapy could help in developing therapeutic targets [24] and also in particularly difficult cases of schizophrenia [25]. Another study found improvements in auditory visual hallucinations, symptoms of depression, and overall quality of life following therapy sessions for treatment-resistant schizophrenia patients [26]. ‘Avatar coaches’ have also been employed as part of an immersive virtual reality situation for treating the fear of heights [27] or as ‘virtual patients’ to provide medical students with lifelike interviewing practice [28]. Finally, avatars are also being implemented in risk prevention education, such as the Kognito program, which uses an avatar to help college students and faculty identify others at risk for suicide [29].

#### Artificially Intelligent Robot Therapy

In addition to these virtually embodied therapeutic applications, clinicians and scientists are exploring the translation of innovations at the intersection of AI and robotics into the clinic. For example, intelligent animal-like robots such as Paro, a fuzzy harp seal, are increasingly being used to help patients with dementia. Paro, along with the large furry eBear, is part of a class of ‘companion bots,’ engaging individuals as at-home health care assistants, responding to speech and movement with dynamic ‘dialog’, or seeking to help elderly, isolated, or depressed patients through companionship and interaction. Several studies have examined the role of such robots in reducing stress, loneliness, and agitation and in improving mood and social connections [30,31]. Thus far, the outcomes are promising [32,33].

AI robots also provide opportunities for different forms of engagement with children suffering from autism spectrum disorders (ASDs) [34]. Children with autism have been found to react positively to robots, even in cases where they have trouble interacting with others [35]. The Kaspar robot has demonstrated potential for integration in current education and therapy interventions [36] and is being investigated for the potential to improve social skills among children [37]. Similarly, RoboTherapy is an example of socially assistive robotics designed to help children with ASDs to develop social skills, and the robot Nao is designed to improve facial recognition and appropriate gaze response. The aim of such robotic interaction is to learn appropriate social skills (eg, imitation, taking turns, staying engaged, and empathy), with the hope that children can then apply the skills learned with the robot peer to their relationships with human peers. Initial studies are promising; individuals with ASDs performed better with their robot partners than human therapists, responded with social behaviors toward robots, and improved spontaneous language during therapy sessions [38]. However, the devices are still being developed and are not yet in wider therapeutic use.

AI-enabled robots are also being explored across a variety of other mental health areas including mood and anxiety disorders, children with disruptive behavior, and patients who may not have a specific diagnosis but who would benefit from assistance with mental health concerns [39]. Perhaps, most controversially, artificially intelligent robots have entered the field of human sexuality. Companies are now offering adult sex robots such as Roxxxy, which can speak, learn their human partners’ preferences, register touch, and provide a form of intimate companionship. Although the range of medical applications that sex robots can reportedly address remains debated, these include meeting the sexual needs of disabled and elderly individuals or as part of therapy for concerns such as erectile dysfunction, premature ejaculation, and anxiety surrounding sex [40]. Furthermore, some researchers have asked if sex robots could help to reduce sex crimes such as rape and assault or be used for treatment of paraphilia, such as pedophilia [3,41].

## Methods

Based on a thematic literature analysis and established principles of medical ethics, an analysis of the ethical and social aspects of currently embodied AI applications was conducted across the fields of Psychiatry, Psychology, and Psychotherapy. To enable a comprehensive evaluation, the analysis was structured around the following three steps: assessment of potential benefits; analysis of overarching ethical issues and concerns; discussion of specific ethical and social issues of the interventions.

## Results

### Ethical and Social Implications and Concerns

The devices and applications described above have yet to be integrated into widespread clinical use. However, in view of the speed of research and development trajectories of these applications, it is reasonable to expect that therapeutic chatbots, avatars, socially assistive devices, and sex robots will soon translate into broader clinical applications in earnest. In some cases, initial ethical assessments are already available [42,43]; however, most of these studies focus on a single application. In general, for most of the applications we are discussing, large-scale rigorous research studies have not yet been conducted or are still in pilot stages [44-47]. Even in Web-based non-AI applications, evidence of patient acceptance and treatment outcomes in routine care is still limited and mixed [48,49]; there has so far been very little research on patient acceptance and contingent treatment outcomes of embodied AI applications in mental health fields. As with any medical innovation, the effects, impacts, and clinical utility of the applications can only be fully assessed once evidence has improved [50,51].

To enable responsible and responsive innovation and clinical translation into the field of mental health, further and more in-depth analysis of the ethical and social implications of embodied AI is necessary to flag areas of concern. Early identification of ethical issues can help researchers, designers, and developers consider these concerns in the design and construction of the next generation of AI agents and robots for mental health. In the following sections, we have provided an analysis of benefits, challenges, and risks of embodied AI in mental health from an ethical perspective. Beginning with a discussion of potential benefits, we have then turned to risks and challenges, followed by immediate concerns in clinical application and long-term effects.

#### Anticipated Benefits

All of the aforementioned intelligent applications promise significant benefits for the field of mental health, satisfying many aspects of the ethical principle of beneficence [52]. From a clinical point of view, the use of embodied AI applications holds the potential to open new avenues for intervention in places where there are still significant unmet health needs. AI interventions might be particularly well placed for detecting mental health concerns early on, for reaching high-risk groups such as veterans, or for those who are concerned about the social stigma associated with psychotherapy [53]. In some cases, patients may respond positively and productively to the fact that the counterpart is *not* a human therapist [54-56]. In one study, subjects overwhelmingly preferred the virtual agent over the human counterpart when being discharged from the hospital because they could self-direct the pace of information—something that is especially important for low-literacy patients [57]. Thus, in mental health services, nonhuman virtual or robotic applications might be preferable for some patients, reducing embarrassment when asking for specific information or services or feelings of shame when admitting noncompliance with a treatment plan. Embodied AI in mental health could also help to empower particular patient groups (such as those who are less familiar with the medical system), thereby helping to improve trust and openness between patients and the medical system. Another important advantage of AI applications is that many of them are low-threshold and self-administered, such that people who do not have an acute condition can elect services without going through the time-consuming process of being screened and admitted into the health care system.

Arguably, the greatest benefit of AI applications is structural, namely the potential to reach populations that are difficult to treat via traditional routes of provision. The provision of some mental health services, for example, through low-threshold, convenient therapeutic interventions via chatbots or avatars may be particularly beneficial for populations living in resource-poor settings. For those living in remote or rural locations or in settings where on-site mental health services are scarce, intelligent applications can increase geographical access and provide some minimal mental health care services where they are otherwise absent. The same may also be true for individuals living in higher income countries who do not have insurance or whose insurance does not cover therapy. Furthermore, it is likely that there are individuals who, for various reasons, do not respond to more traditional clinical services and might prefer low-threshold interventions that can be conducted in the privacy of their homes or on the go. For all of these patients, AI applications could complement existing services or constitute an entry point for pursuing more standard clinical interventions in the future.

In sum, embodied AI interventions may offer entirely new modes of treatment that are potentially more successful than traditional modalities either because they address hard-to-reach populations or because patients respond better to them. Whether, and for which conditions this is the case, requires further investigation. However, given that broadly speaking, conditions such as ASD and sexual dysfunction are increasing in incidence and patient populations with these and many other mental health conditions continue to have unmet health needs [58-62], increased exploration of embodied AI in these fields is promising.

Finally, there are also clear benefits of having a virtual or robotic therapist that is always accessible, has endless amounts of time and patience, never forgets what a patient has said, and does not judge [63,64], thus potentially offering a service that is highly reliable and particularly well-suited to certain patient populations. If integrated into a scaled provision of services, AI-enabled applications could provide support for mild cases of depression and other nonacute conditions [65], therefore helping health professionals to devote more time to the most severe cases. In view of overall increasing burden of illness in mental health and against a background of limited resources, these are important benefits to consider. However, it is likely that embodied AI may not be warmly received by all mental health care professionals, and some may even have serious misgivings about its use because of ethical or clinical concerns. Thus far, there has been no substantial review of the reception of AI across or within specific mental health fields, marking an area in need of further research.

#### Overarching Ethical Concerns

##### Harm Prevention and Data Ethics Issues

To satisfy the well-established ethical principle of nonmaleficence, more robust research is needed on embodied AI applications in mental health to prevent harm both within therapeutic encounters and in cases where robots could malfunction or operate in unpredictable ways. For instance, in interviewing respondents working with AI robotic technologies, Cresswell et al discuss an example of a woman who was stuck in an elevator with a robot and another who was run over by a robot [6]. Chatbots and avatars could also stop working or malfunction. Hence, it needs to be discussed if embodied AI devices—potentially including virtual agents and freely available mental health applications—should require the same kind of rigorous risk assessment and regulatory oversight that other medical devices are subject to before they are approved for clinical use.

Similar to other devices employed in medical settings, the use of any AI applications in mental health care requires careful consideration surrounding data security of devices that communicate personal health information, the ways that the data generated is used, and the potential for hacking and nonauthorized monitoring [66,67]. Clear standards are needed on issues surrounding confidentiality, information privacy, and secure management of data collected by intelligent virtual agents and assistive robots as well as their use for monitoring habits, movement, and other interactions [68,69]. Concerns around privacy may be amplified as the amount of data collected continues to expand; for example, we anticipate that applications that integrate video data would need to have specific privacy protections in place for the communication of sensitive information, or information pertaining to individuals other than the consenting patient.

#### Lack of Guidance on Development, Clinical Integration, and Training

With embodied AI being one of the newest and most rapidly changing areas of psychological and psychiatric research and treatment, existing legal and ethical frameworks are often not closely attuned to these changes. Rather than providing regulatory guidance, there is the risk that the ‘gaps’ between application and ethical frameworks would only be addressed once harm had already occurred [6]. Again, this is the case with many forms of emerging medical technologies; however, in view of the rapid pace of translation of embodied AI into practice in settings where traditional health technology assessment and medical oversight systems are not fully applicable—for example, through freely available therapy chatbots—this is an important concern. Although anticipating the ethical and legal questions that will emerge alongside future developments is difficult, active reflection on the ‘regulatory fit’ for embodied mental health AI is necessary. Initiatives for establishing guidelines are emerging, including the online collaboratively generated document “Moral Responsibility for Computing Artifacts: The Rules,” or the recent “An Ethical Framework for a Good AI Society: Opportunities, Risks, Principles, and Recommendations” [70]. However, thus far, no guidance exists that is specific to the field of mental health services; pointing to the need for the development of further recommendations to better guide advances in this area.

In addition to a lack of guidance on the development of these interventions for design, use, and regulatory questions, so far, there are also no frameworks available on how medical professionals can effectively engage with and train for increased use of embodied AI in the clinic; that is, although there is an increasing body of both academic and popular literature on how embodied AI can be integrated into clinical practice in mental health, there remains a lack of high-level guidance from professional bodies on the best use of AI in mental health services [15,71-73]. There are also no recommendations available on how to train and prepare young doctors for a mental health field in which such tools will increasingly be used by patients. Thus, further ethical guidelines are needed that are specific to assisting mental health professionals who will be supervising patients who have, or possibly will, engage with AI services.

### Potential for Misuse to Reduce Service Provision

An ethically informed integration of AI should also consider questions of a just provision of mental health care [52]. There is the worry that the incorporation of embodied AI in mental health could be a justification for replacing established services, resulting in fewer available health resources or principally AI-driven services, thereby potentially exacerbating existing health inequalities. Many proponents insist that although informed by evidence-based psychotherapeutic approaches, chatbots, for instance, are not intended to replace therapists entirely. In some cases, forms of ‘blended’ care involving both in-person and virtual forms of therapy are being explored [74], which might also be appropriate for intelligent applications. Blended care models potentially offer the opportunity to draw on the strengths of both AI applications and in-person clinical supervision. However, whether or not it is appropriate to implement AI applications in mental health care depends in part on the availability of other resources in that area. As noted, in cases with limited mental health services, AI applications could provide a needed resource that is decidedly better than no services at all. However, at this point, AI mental health services are not a substitute or a stand-in for the kind of robust, multitiered mental health care available in high-resource health care systems. Appropriately considering the status quo of mental health resources in each context is thus highly relevant from an ethical perspective [75]. Otherwise, AI tools in mental health could be used as an excuse for reducing the provision of high-quality, multilayered care by trained mental health professionals in low-resource settings.

## Discussion

### Specific Challenges in Application

#### Risk-Assessment, Referrals, and Supervision

Considering the application of embodied AI tools in mental health practice, a host of specific challenges need to be kept in mind: mental health professionals have an ethical responsibility to inform other service providers as well as third parties or authorities if a patient indicates that they are a threat to themselves or to another individual. How this would work in artificially intelligent interventions, particularly when there is no supervision of the interaction between the AI agent and the patient by a qualified health professional, remains to be determined. It is unclear when, and how, assistive robots that patients have in their homes, or freely available virtual agents and chatbots, would effectively connect at-risk individuals with appropriate services, including hospitalization and other protections. This scenario is particularly relevant in the aforementioned situation of using AI mental health applications to extend access to rural, hard to reach, or uninsured populations. In these cases, some provision of service is arguably better than nothing. However, what should be done if, for example, a therapy bot detects through speech patterns that an individual is at higher risk for self-harm, yet appropriate referral services are not available in the area?

AI applications engaged in therapeutic relationships with clients will likely also need to be bound by similar ethical guidelines as those that bind mental health professionals. However, so far, how an AI duty of care or a code of practice on reporting harm should be operationalized is entirely unclear. An obvious suggestion would be to always mandate supervision through a qualified mental health clinician—when a human therapist evaluates a patient’s expression of self-harm, she also considers contextual information in her interpretation of the level of risk. Whether, and to what degree, robotic therapists are able to do this remains unclear. However, many AI applications are available outside established mental health settings; in addition, the capacity of computerized methods to identify and predict psychiatric illness are increasing [12,13], as are their skills of therapeutic interaction and communication. Thus, the question of whether supervision of embodied AI in health should always be provided, and how such a requirement could be successfully implemented, remains a subject for further debate.

### Respecting and Protecting Patient Autonomy

Another concern for the application of embodied AI in mental health practice centers on enabling and respecting patient autonomy [52]. These are novel technologies that require assessment to guarantee that patients fully understand how the application or avatar works in order to ensure that a patient does not misunderstand or mistake the intelligent system for a human-driven application.  For instance, it would be problematic if a patient were to assume that ‘at the other end’ of the chatbot there is a doctor communicating or reviewing her messages. Furthermore, obtaining consent for applications used outside of medical systems raises thorny concerns. For instance, an elderly person or a person with intellectual disabilities may not be able to understand what a robot is or what a robot does when it is installed at home to monitor the patient’s activities, risking privacy infringement [42], manipulation, and even coercion if the conditions for informed consent are not satisfied. Such questions make consenting to surveillance, interaction, and data collection with the robot challenging matters. Distinctions could be drawn between interventions that are seen as helping and monitoring, as opposed to those that run the risk of manipulation and coercion; however, these lines are often blurred both in theory and in practice [76]. How AI applications should evaluate if a patient has fully understood the information provided when giving consent, and how to proceed in cases where it is not possible for individuals to provide consent, such as children, patients with dementia, those with intellectual disabilities, or those in acute phases of schizophrenia, needs to be addressed.

Another area of particular concern in relation to matters of promoting autonomy in the use of AI in mental health care is the engagement of vulnerable populations. People have been shown to be more compliant when a robot asks them to do something as compared with a person [9]. Although this could lead to better results when helping patients with autism or those needing to make difficult behavioral changes, the concern exists that people could be manipulated or coerced into doing things that they should not or that they have not fully thought through, either because of the novelty of the device or because of a lack of companions with whom to discuss alternatives. Some studies have made a distinction between a “suspension of disbelief” when anthropomorphizing a robot caregiver and deception *per se* [42,77], but this is a line that requires further investigation in practice.

### Nonhuman Therapy?

In general, the question remains as to whether there are aspects of the therapeutic encounter that cannot be achieved through AI. Some therapeutic benefits may be difficult to anticipate, or highly specific to a particular individual’s relationship to his or her therapist. One study found that embodied conversational agents had difficulty evaluating a user’s emotional state in a real-time dialog and that the absence of a human therapist in Web-based mental health interventions for treating depression and anxiety had a negative effect on user adherence to the programs [78]. In the treatment of insomnia, some patients indicated that they missed having a human therapist [79]. Relatedly, because robots and artificially intelligent systems blur previously assumed boundaries between reality and fiction, this could have complex effects on patients. Similar to therapeutic relationships, there is the risk of transference of emotions, thoughts, and feelings to the robot. In particular, given that many of the target populations are vulnerable because of their illness, age, or living situation in a health care facility, there is the additional concern that patients would be vulnerable in their engagements with the robot because of their desire for company or to feel cared for [80]. Unlike with a therapist, however, there is no person on the other side of this transference. Whether robot therapists will ever be able to deal adequately with such transference remains to be seen. Further concerns are likely to emerge in practice; thus, embodied AI therapeutic aids need to also be evaluated carefully for unanticipated differences with standard therapy modalities.

### Ethical Issues in Algorithms

It is necessary to note that AI mental health interventions work with algorithms, and algorithms come with ethical issues. It has been well-established that existing human biases can be built into algorithms, reinforcing existing forms of social inequality [81]. This raises the concern that AI-enabled mental health devices could also contain biases that have the potential to exclude or harm in unintended ways, such as data-driven sexist or racist bias or bias produced by competing goals or endpoints of devices [82,83]. Following other calls for transparency [84], the algorithms used in artificially intelligent applications for mental health purposes could be similarly open to scrutiny. This may require investing additional time in explaining to patients (and their families) what an algorithm is and how it works in relation to the therapy provided [85]. However, how to best do this, in particular with patients with compromised mental capacities, requires further consideration.

### Concerns Regarding Long-Term Effects

Apart from these more immediate concerns, the implementation of embodied AI into mental health services also raises a number of broader questions regarding long-term impacts on patients, the mental health community, and society more widely. For instance, it has been noted that long-term use of AI interventions could lead to some patients or patient groups becoming overly attached to these applications. A study by Cresswell et al noted that robots that aim to alleviate loneliness or provide emotional comfort carry the risk that the patients they work with could become dependent on them [6]. More broadly, others have raised questions about ways that robots could contribute to changing social values surrounding care or situations in which caregiving is increasingly ‘outsourced’ to robotic aids. The impact of intelligent robots on relationships, both human-robot and human-human relationships, is an area that requires further probing, as do potential effects on identity, agency, and self-consciousness in individual patients. Specifically, research into the effectiveness of these applications needs to cover not only if the social skills of children with ASD are improved by working with robots but also their ability to apply these skills to relationships with other humans. Similarly, if a sex robot is provided therapeutically to an individual with paraphilia, the effects of this on the targeted behaviors with other humans also needs to be evaluated. The risk exists that if robotic interventions are not translatable to improving human interaction, that they merely remain a way of improving human relations with machines, or worse, an outlet that further limits human-to-human relationships. Similarly, engagement with embodied intelligent devices could also have important effects on the individual, such as on personal sense of identity or agency.

The integration of AI devices into our everyday lives and medical care is undoubtedly changing social expectations and practices of communication. There are essential differences between communicating with an AI device and communicating with another human. Anecdotal findings suggest that some users often speak to assistive devices such as Siri or Alexa in a curter or ruder manner than they would to a human [86]. Importantly, perceptions of the devices can vary by users: children often understand these devices differently than adults, sometimes attributing human characteristics to the device or believing that the device has a real individual inside [87]. Extrapolating from this example, it is clear that the ways that individuals interact with the AI applications in their lives can have implications for communication and social interaction. How this will evolve as more patients have the opportunity to interact with AI applications as part of their mental health care requires further empirical investigation to catch problematic trends early and correct for future development.

A related concern of objectification exists for some areas of AI applications, such as sex robots. The use of ‘sexbots’ has already been notably controversial, with scholars objecting that sexual dysfunction depends on a range of physical, psychological, and sociocultural factors that are profoundly relational and reciprocal. Rather than addressing issues of isolation associated with sexual dysfunction, robots might aggravate it or contribute to reductionist understandings of sexual violence [88]. It has been cautioned that the use of sex robots—also available in childlike models or programed with personalities such as “Frigid Farrah” to resist sexual advances—could instead increase the occurrence of sex crimes, normalize the production of social inequalities surrounding the male gaze [89], and contribute to unwanted sexual encounters. Furthermore, the creation of humanoid robots for use in sexual dysfunction raises concerns that it could reinforce or even legitimize the objectification of humans, in particular women and children [3,88]. As the use of AI in many therapeutic applications has not yet been validated in randomized controlled trials (RCTs), there is the risk that particular applications might make problems such as sexual violence worse. More broadly, embodied AI applications necessarily involve a relatively narrow understanding of illness. For instance, sex robots may help with some medical concerns but do not address other determinants of illness that would have to be taken into account from a bio-psycho-social understanding of mental health illness. Widespread AI use could thus exacerbate trends of reductionism in mental health.

Ideas around embodied AI are culturally and historically shaped. Whether providing motivational interviews in therapy [64], acting as embodied conversational agents for mental disorders [47], or working with populations with intellectual disabilities [90], discussion of embodied AI often turns to worries surrounding the limits of human control over technology. Conjuring images of the Terminator or other depictions of the nonhuman in science fiction or cinema, such tools can carry with them negative or scary associations that bring the issue of trust in medical practice into new light [6]. However, exposure to robotic devices, or living in places with positive or caring associations with robots, can influence the adoption of AI devices in different settings [91]. Initiatives that integrate embodied AI into health care practices need to be duly attuned to existing cultural understandings of the role of technology in social lives, and work to ensure that trust between patient and provider, or patient and the health care system, is not eroded.

Finally, AI agents for mental health raise fundamental questions about what it is to be human [6]. One of the principal contributions of science and technology studies scholarship has been to show how humans do not simply act upon objects but rather our relationships with objects also alters, transforms, and imposes limits upon human activity [92]. Interaction with embodied AI agents, just like interaction with other individuals or a therapist, alters behaviors and understandings of the world. Although social relationships are characterized by reciprocity, relationships with intelligent devices are neither mutual nor symmetric. In particular, some have raised the concern that interacting more with artificial agents may lead some individuals to engage less with other people around them or to develop forms of intimacy with intelligent robots [93], raising concerns specific to the use of robots with children or those with intellectual disabilities. As mentioned, people develop attachments to objects and have been shown to also develop attachments to simpler robotic systems such as AIBO. Thus, it is likely that as more intelligent and autonomous devices are developed, human relationships with them will become even more complicated [94].

### Conclusions

In light of the demonstrated benefits and potential, such as expanding the reach of services to underserved populations or enhancing existing services provided by mental health professionals, embodied AI has emerged as an exciting and promising approach across the field of mental health. At present, the quality of research on embodied AI in psychiatry, psychology, and psychotherapy is varied, and there is a marked need for more robust studies including RCTs on the benefits and potential harms of current and future applications.

This is still an emerging field, and any analysis of ethical implications can only be preliminary at this point. However, a few conclusions and recommendations are warranted, based on the considerations presented in this paper:

1. It is necessary to develop clear guidance on whether (and which) embodied AI applications should be subject to standard health technology assessment and require regulatory approval. This should include a set of broader provisions for the use of AI services outside the supervision of a health care professional.
2. Professional associations in mental health should develop guidelines on the best use of AI in mental health services as well as recommendations on how to train and prepare young doctors for wide-spread use of embodied AI in mental health, including blended care models.
3. AI tools in mental health should be treated as an additional resource in mental health services. They should not be used as an excuse for reducing the provision of high-quality care by trained mental health professionals, and their effect on the availability and use of existing mental health care services will need to be assessed.
4. To satisfy duties of care and reporting of harm, ideally embodied AI should remain under the supervision of trained mental health professionals. Any applications offered outside of mental health care settings, such as apps and bots, should be required to demonstrate reliable pathways of risk-assessment and referral to appropriate services.
5. Embodied AI should be used transparently. Guidance on how to implement applications in a way that respects patient autonomy needs to be developed, for example, regarding when and how consent is required and how to best deal with matters of vulnerability, manipulation, coercion, and privacy.
6. AI algorithms in mental health need to be scrutinized, for example, for bias. Ideally, health professionals should be trained in communicating to their patients the role of the algorithms used in different applications they might be using or consider using, and such algorithms should be open for public debate and shaping.
7. Increased use of embodied AI should be accompanied by research that investigates both direct and indirect effects on the therapeutic relationship, other human-human relationships, and effects on individual self-consciousness, agency, and identity. Long-term effects, ranging from health reductionism to increased objectification and impacts on our understandings of what it means to be human, need to be monitored.

## Abbreviations

AI: artificial intelligence
ASD: autism spectrum disorder
RCT: randomized controlled trial
